# Supplementary material for: The influence of preprocessing on text classification using a bag-of-words representation
Source: PLoS One. 2020 May 1;15(5):e0232525. doi: 10.1371/journal.pone.0232525 (PMC7194364; doi:10.1371/journal.pone.0232525)
Supplement: S1 Appendix — (DOCX) [file pone.0232525.s001.docx]

Appendix

This appendix includes two examples of files from each of these four datasets.

**WebKB – Example # 1:**

MIME-Version: 1.0

Server: CERN/3.0

Date: Wednesday, 20-Nov-96 20:17:51 GMT

Content-Type: text/html

Content-Length: 1946

Last-Modified: Thursday, 14-Nov-96 17:42:20 GMT

<html> <head>

<title>CS414 Home Page</title>

</head>

<body>

<center><img src = "Icons/cs414.gif"></center>

<center><h2>CS414: Systems Programming and Operating Systems</h1></center>

<center><h2>

<a href = "cs415.html">CS415 Practicum in Operating System</a>

</h2></center>

<center><h2>Kenneth P. Birman</h2></center>

<hr>

<h3><a href = "news://newsstand.cit.cornell.edu/cornell.class.cs414">CS414/415 News Group</a></h3>

<h3><a href = "syllabus.ps">Course Syllabus</a></h3>

<h3>Lecture Notes</h3>

<ul>

<li><a href = "filesys.ps">Unix Filesystem Structure</a>

<li><a href = "linking.ps">Linking (Static and Dynamic)</a>

</ul>

<h3>Assignments</h3>

<ul>

<li><a href = "hw1.ps">Assignment 1</a>

<li><a href = "hw2.ps">Assignment 2</a>

<li><a href = "hw3.ps">Assignment 3</a>

<li><a href = "hw4.ps">Assignment 4</a>

</ul>

<h3>Assignment Solutions</h3>

<ul>

<li><a href = "sol1.ps">Solution 1</a>

<li><a href = "sol2.ps">Solution 2</a>

<li><a href = "sol4.ps">Solution 4</a>

<li><a href = "sol5.ps">Solution 5</a>

</ul>

<h3><a href = "prelim1-soln.ps">Prelim 1 Solution</a></h3>

<!--

<h3><a href = "p1-s.prn">Prelim 1 Solution</a></h3>

-->

<h3>TAs</h3>

<ul>

<li>LiLi<br>

5162 Upson Hall<br>

Phone: 255-7421<br>

E-Mail: <a href="mailto:lili@cs.cornell.edu">

lili@cs.cornell.edu</a><br>

Office Hours: Wednesday and Friday 3:30-5:00

<li>Yi-Cheng Huang<br>

5151 Upson Hall<br>

Phone: 255-3042<br>

E-Mail: <a href="mailto:ychuang@cs.cornell.edu">

ychuang@cs.cornell.edu</a><br>

Office Hours: Tuesday and Thursday 2:00-3:30

<li>Mihai Budiu<br>

4132 Upson Hall<br>

Phone: 255-1179<br>

E-Mail: <a href="mailto:budiu@cs.cornell.edu">

budiu@cs.cornell.edu</a><br>

Office Hours: Wednesday 11:00-12:30 and Thursday 11:30-1:00

</ul>

<hr>

<address></address>

<!-- hhmts start -->

Last modified: Thu Nov 14 12:42:19 1996

<!-- hhmts end -->

</body> </html>

**WebKB – Example # 2:**

Date: Thu, 21 Nov 1996 22:53:38 GMT

Server: NCSA/1.4.2

Content-type: text/html

Last-modified: Thu, 10 Oct 1996 16:31:01 GMT

Content-length: 126

<html>

<title> Jovan's Home Page </title>

Jovan's home page moved to <A HREF="http://www.cs.cmu.edu/~jovan"> CMU</A>.

</html>

**R8 – Example # 1:**

chemlawn chem rises on hopes for higher bids chemlawn corp chem could attract a higher bid than the dlrs per share offered by waste management inc wnx wall street arbitrageurs said shares of chemlawn shot up to in over the counter trading with mln of the company s mln shares changing hands by late afternoon this company could go for times cash flow or dlrs maybe dollars depending on whether there is a competing bidder an arbitrageur said waste management s tender offer announced before the opening today expires march this is totally by surprise said debra strohmaier a chemlawn spokeswoman the company s board held a regularly scheduled meeting today and was discussing the waste management announcement she said a statement was expected but it was not certain when it would be ready she was unable to say if there had been any prior contact between waste management and chemlawn officials i think they will resist it said elliott schlang analyst at prescott ball and turben inc any company that doesn t like a surprise attack would arbitrageurs pointed out it is difficult to resist tender offers for any and all shares for cash schlang said chemlawn could try to find a white knight if does not want to be acquired by waste management analyst rosemarie morbelli of ingalls and snyder said servicemaster companies l p svm or rollins inc rol were examples of companies that could be interested chemlawn with about two mln customers is the largest u s company involved in application of fertilizers pesticides and herbicides on lawns waste management is involved in removal of wastes schlang said chemlawn s customer base could be valuable to another company that wants to capitalize on a strong residential and commercial distribution system both schlang and morbelli noted that high growth rates had catapulted chemlawn s share price into the mid s in but the stock languished as the rate of growth slowed schlang said the company s profits are concentrated in the fourth quarter in chemlawn earned dlrs per share for the full year and dlrs in the fourth quarter morbelli noted chemlawn competes with thousands of individual entrepreuers who offer lawn and garden care sevice reuter

**R8 – Example # 2:**

dean foods df sees strong th qtr earnings dean foods co expects earnings for the fourth quarter ending may to exceed those of the same year ago period chairman kenneth douglas told analysts in the fiscal fourth quarter the food processor reported earnings of cts a share douglas also said the year s sales should exceed billion dlrs up from billion dlrs the prior year he repeated an earlier projection that third quarter earnings will probably be off slightly from last year s cts a share falling in the range of cts to cts a share douglas said it was too early to project whether the anticipated fourth quarter performance would be enough for us to exceed the prior year s overall earnings of dlrs a share in douglas said dean should experience a pct improvement in our bottom line from effects of the tax reform act alone president howard dean said in fiscal the company will derive benefits of various dairy and frozen vegetable acquisitions from ryan milk to the larsen co dean also said the company will benefit from its acquisition in late december of elgin blenders inc west chicago he said the company is a major shareholder of e b i foods ltd a united kingdom blender and has licensing arrangements in australia canada brazil and japan it provides ann entry to mcdonalds corp mcd we ve been after for years douglas told analysts reuter

**SMS Spam Collection – Example # 1:**

Go until jurong point, crazy.. Available only in bugis n great world la e buffet... Cine there got amore wat...

**SMS Spam Collection – Example # 2:**

WINNER!! As a valued network customer you have been selected to receivea £900 prize reward! To claim call 09061701461. Claim code KL341. Valid 12 hours only.

**Sentiment Labelled Sentences – Example # 1:**

So there is no way for me to plug it in here in the US unless I go by a converter.

**Sentiment Labelled Sentences – Example # 2:**

Good case, Excellent value.
