# Supplementary material for: The influence of preprocessing on text classification using a bag-of-words representation
Source: PLoS One. 2020 May 1;15(5):e0232525. doi: 10.1371/journal.pone.0232525 (PMC7194364; doi:10.1371/journal.pone.0232525)
Supplement: Attachment — Submitted filename: Our responses to Reviewers comments-Third-Version-22.3.20.docx [file pone.0232525.s003.docx]

מאת: **PLOS ONE** <em@editorialmanager.com>
Date: יום ה׳, 19 במרץ 2020 ב-19:12‬
Subject: PLOS ONE Decision: Revision required [PONE-D-19-27170R1] - [EMID:72e8a1c6a74673ac]
To: Yaakov HaCohen-Kerner <kerner@jct.ac.il>‬‬‬

PONE-D-19-27170R1
The Influence of Preprocessing on Text Classification using a Bag-of-Words Representation
PLOS ONE

Dear Prof. HaCohen-Kerner,

Thank you for submitting your manuscript to PLOS ONE. After careful consideration, we feel that it has merit but does not fully meet PLOS ONE’s publication criteria as it currently stands. Therefore, we invite you to submit a revised version of the manuscript that addresses the points raised during the review process.
==============================
I have read the two rounds of reviews and the authors' responses to the orignal review.

As indicated by Reviewer #2, we can see the efforts made by the authors as well as their difficulties.

However, there are some questions that need to be clarified and explained.

Please refer to the reviews raised by Reviewer #2. Thanks very much.

==============================

We would appreciate receiving your revised manuscript by May 03 2020 11:59PM. When you are ready to submit your revision, log on to <https://www.editorialmanager.com/pone/> and select the 'Submissions Needing Revision' folder to locate your manuscript file.

If you would like to make changes to your financial disclosure, please include your updated statement in your cover letter.

To enhance the reproducibility of your results, we recommend that if applicable you deposit your laboratory protocols in [protocols.io](http://protocols.io), where a protocol can be assigned its own identifier (DOI) such that it can be cited independently in the future. For instructions see: <http://journals.plos.org/plosone/s/submission-guidelines#loc-laboratory-protocols>

Please include the following items when submitting your revised manuscript:

- A rebuttal letter that responds to each point raised by the academic editor and reviewer(s). This letter should be uploaded as separate file and labeled 'Response to Reviewers'.
- A marked-up copy of your manuscript that highlights changes made to the original version. This file should be uploaded as separate file and labeled 'Revised Manuscript with Track Changes'.
- An unmarked version of your revised paper without tracked changes. This file should be uploaded as separate file and labeled 'Manuscript'.

Please note while forming your response, if your article is accepted, you may have the opportunity to make the peer review history publicly available. The record will include editor decision letters (with reviews) and your responses to reviewer comments. If eligible, we will contact you to opt in or out.

We look forward to receiving your revised manuscript.
Kind regards,
Weinan Zhang
Academic Editor
PLOS ONE

[Note: HTML markup is below. Please do not edit.]

Dear Dr. Weinan Zhang, Dear reviewers,

Thank you very much for offering us the opportunity to revise and resubmit our work. We much appreciate the insightful comments from the two respected reviewers for the previous versions of this paper. Their concerns have been addressed in this new manuscript, and our paper has been revised and improved. Please see below our point-by-point response to each of the comments.

Thank you for your consideration of our work.

Sincerely,

Yaakov HaCohen-Kerner, Daniel Miller, and Yair Yigal

Reviewers' comments:
Reviewer's Responses to Questions
**Comments to the Author**

1. If the authors have adequately addressed your comments raised in a previous round of review and you feel that this manuscript is now acceptable for publication, you may indicate that here to bypass the “Comments to the Author” section, enter your conflict of interest statement in the “Confidential to Editor” section, and submit your "Accept" recommendation.

Reviewer #1: All comments have been addressed
Reviewer #2: (No Response)

2. Is the manuscript technically sound, and do the data support the conclusions?

The manuscript must describe a technically sound piece of scientific research with data that supports the conclusions. Experiments must have been conducted rigorously, with appropriate controls, replication, and sample sizes. The conclusions must be drawn appropriately based on the data presented.

Reviewer #1: Yes
Reviewer #2: Yes

3. Has the statistical analysis been performed appropriately and rigorously?

Reviewer #1: Yes
Reviewer #2: Yes

4. Have the authors made all data underlying the findings in their manuscript fully available?

The [PLOS Data policy](http://www.plosone.org/static/policies.action#sharing) requires authors to make all data underlying the findings described in their manuscript fully available without restriction, with rare exception (please refer to the Data Availability Statement in the manuscript PDF file). The data should be provided as part of the manuscript or its supporting information, or deposited to a public repository. For example, in addition to summary statistics, the data points behind means, medians and variance measures should be available. If there are restrictions on publicly sharing data—e.g. participant privacy or use of data from a third party—those must be specified.

Reviewer #1: No
Reviewer #2: No

**5. Is the manuscript presented in an intelligible fashion and written in standard English?**

PLOS ONE does not copyedit accepted manuscripts, so the language in submitted articles must be clear, correct, and unambiguous. Any typographical or grammatical errors should be corrected at revision, so please note any specific errors here.
Reviewer #1: No
Reviewer #2: No

**6. Review Comments to the Author**

Please use the space provided to explain your answers to the questions above. You may also include additional comments for the author, including concerns about dual publication, research ethics, or publication ethics. (Please upload your review as an attachment if it exceeds 20,000 characters)

**Reviewer #1:** The paper is very interesting and the analysis results very useful. The authors tackled all changes, and thus, the paper is worth for publishing.

**Our response:** Thank you very much for all your helpful and clever comments.

**Reviewer #2:** I understand that it is difficult to continue the research when the students complete their program and start new jobs. Nevertheless, it is very important to maintain a backup of all data used/generated during the experimental evaluations.

**Our response:** All the computers in the graduation projects lab were reset and all the files in them were deleted. We will endeavor to learn a lesson and do so in subsequent studies.

The revision and the explanations added to the revised version of the paper improved its quality. The points that still need some attention are:

**Our response:** Thank you very much for all your helpful and clever comments.

1. Re-write the last paragraph of the Introduction, describing the Sections of the paper.

**Our response:** Done.

2. Provide the section Materials and Methods, which were included in the revised version without content.

**Our response:** Done.

3. Example #1 of WebKB (Appendix) seems to be only the content of an HTML source. It does not have the HTML tags as Example #2.

**Our response:** We worked with the original documents, which include HTML tags. We replaced the Example #1 in its original file with its HTML tags.

4. About the R8 dataset. The examples of R8 files in the Appendix do not present upper case letters and punctuation. It seems that they are preprocessed files. Is that the case of every file of this dataset used in the experiments? Besides, in Table 8 we can see that P (removing punctuation) and L (converting to lowercase) presented the same accuracy of the baseline. This result may indicate that P and L did not make any effect since the baseline documents are already in lowercase and without punctuation. However, some of the best results for this dataset were obtained with the combination LP. How this could be explained?

**Our response:** This is true. The documents of R8 do not contain upper case letters and punctuation symbols. Therefore, there should be no changes in the results in Table 8 following the application of the L and P preprocessing methods. One of my students checked this matter and he wrote me that what happened is probably because the codes of several combinations of preprocessing methods in several rows of results in Table 8 have been mistakenly replaced. The document "The Influence of Preprocessing on TC-WithChanges" presents corrections, which have been made in Table 8 because of your remark. In some cases, small changes versus expected results occur due to random components.

5. Delete the word “twice” in the sentence “The best result in Table 10, 78.78%v was obtained twice by the RF method using CL”.

**Our response:** Corrected.

6. In the sentence “The majority of the improvement is because of the C preprocessing, which presents a significant improvement of 2.23% from the baseline”, according to Table 10, it is L instead of C.

**Our response:** Corrected. We changed the beginning of the relevant paragraph

from

"The best result in Table 10, 78.78% was obtained by the RF method using CL. The majority of the improvement is because of the C preprocessing, which presents a significant improvement of 2.23% from the baseline;"

to

"The best result in Table 10, 78.78%v (a significant improvement of 2.79% from the baseline) was obtained by the RF method using CL. The majority of the improvement is because of the L preprocessing, which presents a significant improvement of 2.23% from the baseline"

7. PLOS authors have the option to publish the peer review history of their article ([what does this mean?](https://journals.plos.org/plosone/s/editorial-and-peer-review-process#loc-peer-review-history)). If published, this will include your full peer review and any attached files.

If you choose “no”, your identity will remain anonymous but your review may still be made public.

**Do you want your identity to be public for this peer review?** For information about this choice, including consent withdrawal, please see our [Privacy Policy](https://www.plos.org/privacy-policy).

Reviewer #1: No
Reviewer #2: No

[NOTE: If reviewer comments were submitted as an attachment file, they will be attached to this email and accessible via the submission site. Please log into your account, locate the manuscript record, and check for the action link "View Attachments". If this link does not appear, there are no attachment files to be viewed.]

While revising your submission, please upload your figure files to the Preflight Analysis and Conversion Engine (PACE) digital diagnostic tool, <https://pacev2.apexcovantage.com/>. PACE helps ensure that figures meet PLOS requirements. To use PACE, you must first register as a user. Registration is free. Then, login and navigate to the UPLOAD tab, where you will find detailed instructions on how to use the tool. If you encounter any issues or have any questions when using PACE, please email us at [figures@plos.org](mailto:figures@plos.org). Please note that Supporting Information files do not need this step.
